# Improving multi-pinhole CZT myocardial perfusion imaging specificity without changing sensibility by using adapted filter parameters

**DOI:** 10.1186/s13550-024-01083-9

**Published:** 2024-03-07

**Authors:** Fabien Vauchot, Julien Dubois, Aurélie Bourdon

**Affiliations:** 1 Department of Nuclear Medicine, Montpellier University Hospital, Montpellier, France; 2 Department of Radiopharmacy, Montpellier University Hospital, Montpellier University, Montpellier, France

**Keywords:** Myocardial perfusion imaging (MPI), Image artefact, Cadmium-zinc-telluride (CZT) camera, Butterworth filters

## Abstract

**Background:**

Meta-analysis show the diagnostic performance of cardiac dedicated multi-pinhole cadmium-zinc-telluride myocardial perfusion imaging (MPI) with a sensibility around 0.9 and a specificity around 0.7. The aim of the present study is to explore a simple method to generate less artefact on MPI using single photon emission computed tomography (SPECT) and to enhance specificity without changing sensibility.

**Results:**

From October 2018 to March 2019, 200 patients who underwent SPECT with [^99m^Tc]Tc-tetrofosmin were prospectively recruited: 100 patients with ischemia or necrosis diagnosis (first arm), and 100 patients with myocardial reversible SPECT artefact (second arm). Each SPECT was explored using two image process based on a Butterworth prefilter and post-filter: the original image processing (reconstruction A) with a cut-off frequency equals to 37% of the Nyquist frequency and order equals to 7, and a second image processing (reconstruction B) with a cut-off frequency equals to 25% of the Nyquist frequency and order equals to 5. For each patient, sum stress or rest score with and without septum (SSRS and SSRSws) were calculated with the two reconstructions. No significant statistical difference between SSRSa and SSRSb was identified for the first arm (*P* = 0.54) and the relative difference ∆r was − 0.5 ± 11.1% (95% CI − 2.7 to 1.7). We found a significant statistical difference between SSRSa and SSRSb for the second arm (*p* < 0.0001) and the relative difference ∆r was 69.7 ± 16.2% (95% CI 66.6–72.9).

**Conclusion:**

In conclusion, using a Butterworth prefilter and post-filter cut-off frequency equal to 25% of the Nyquist frequency before iterative reconstruction generates less artefact and improves myocardial SPECT specificity without affecting sensibility compared with the original reconstruction.

## Background

The semiconductor cadmium-zinc-telluride (CZT) Discovery NM530c camera (GE Healthcare) is based on stationary multi-pinhole collimation system with a dedicated three-dimensional iterative reconstruction algorithm, which is based on maximum likelihood expectation maximization [1, 2]. Image quality with CZT camera is improved by many factors including: increase in energy resolution, higher spatial resolution and contrast-to-noise compared to conventional Anger camera [3, 4]. The stress myocardial perfusion imaging (MPI) using CZT technology for the diagnosis of coronary artery disease has satisfactory sensitivity but suboptimal specificity [5]. Meta-Analysis indicates a sensibility of 0.84 (95% confidence interval, 95% CI 0.78–0.89) and a specificity of 0.69 (95% CI 0.62–0.76) [6]. Another meta-analysis compares the diagnostic performance of conventional single photon-emission computed tomography (c-SPECT) and CZT-SPECT: the sensibility is 0.89 for CZT-SPECT versus 0.85 for c-SPECT and the specificity 0.69 for CZT camera versus 0.66 for c-SPECT [7]. A new protocol of MPI in CZT-SPECT allows faster exams with less radiation dose and shows similar prognostic results compared to those obtained with a conventional camera [8].

In MPI, soft-tissue attenuation by breasts, lateral chest wall and abdomen may create artefacts that mimic true perfusion defects. Some studies conclude that patients with body mass index (BMI) higher than forty should be scheduled for MPI on c-SPECT because of major artefacts [9]. For the CZT camera, the extent of attenuation artefact is significantly larger compared to conventional camera (23 ± 5% versus 15 ± 5%) [10]. Attenuation correction significantly decreases mean summed stress scores (SSS) and mean summed rest scores (SRS) in MPI with a CZT camera, improves the specificity of MPI with a CZT camera, and lowers the need for additional rest imaging in stress-first MPI, decreasing the mean effective dose [11–13]. Understanding the attenuation pattern in MPI studies using CZT gamma cameras is crucial for physicians who do not use attenuation correction to prevent incorrect image interpretation [12]. Other MPI artefact sources are patient motion (PM) and cardiac respiratory motion (RM). The impact on myocardial perfusion defect appears when ${\text{RM}} > 10\,\,{\text{mm}}$. When ${\text{RM}} > 15\,{\text{mm}}$, myocardial perfusion defects concern 14% of the CZT-SPECT [14, 15]. A study on motion correction concluded that RM have an influence on the diagnostic result, but not PM [16]. Both attenuation correction and motion correction are feasible and increase specificity and frequency of normal scans for MPI using CZT-SPECT [17]. Another classical source of artefact is the incorrect patient positioning in MPI using CZT-SPECT. The SSS and SRS can increase by more than two units for 35% of SPECT when the heart is positioned at the limit of the field of view allowing images of clinical quality [18].

In this study, we investigated an original method to improve specificity while generating less artefact on MPI without changing sensibility when using CZT-SPECT. The aim was to prevent false positive diagnostics, and avoid repeated acquisition to optimize patient management with an ideal goal to have only two acquisitions, one for the rest and one for the stress.

## Methods

From October 2018 to February 2019, we prospectively analysed all 727 patients explored for MPI in our hospital. These scintigraphies were performed with a cardiac dedicated multi-pinhole CZT (Discovery NM 530c, GE Healthcare) gamma camera. Each patient was explored twice the same day with SPECT gated acquisitions: the first acquisition concerned the left myocardial perfusion and functional rest parameters; the second acquisition concerned the same parameters after stress. Only patients exceeding a BMI of 35 were explored in two days. Acquisitions at rest were performed 20 min after 2.6 MBq.kg^−1^ of [^99m^Tc]Tc-tetrofosmin injection. Stress acquisition were performed 5 min after the end of the cardiac stress testing with dipyridamole infusion and injection of 8 MBq.kg^−1^ of [^99m^Tc]Tc-tetrofosmin at the stress peak. The two acquisitions lasted 5 min, in prone position with arms over the head using CZT camera with the following acquisition parameters: list mode, 32 × 32 matrix size, 4 mm pixel size. The left ventricular relative radiotracer uptake of [^99m^Tc]Tc-tetrofosmin upon 17 segments was analysed for each scintigraphy selected, and a numerical value was assigned to each segment based on the relative uptake (RU): 0 ($100 < {\text{RU}} \le 70$); 1 ($70 < {\text{RU}} \le 50$); 2 ($50 < {\text{RU}} \le 30$); 3 ($30 < {\text{RU}} \le 10$); 4 (${\text{RU}} < 10$). Summed stress or rest score (SSRS) was calculated by addition of the 17 values. A second SSRS without septal component was calculated (excluding septo-basal and infero-septo-basal segments) named SSRSws.

**Fig. 1:**
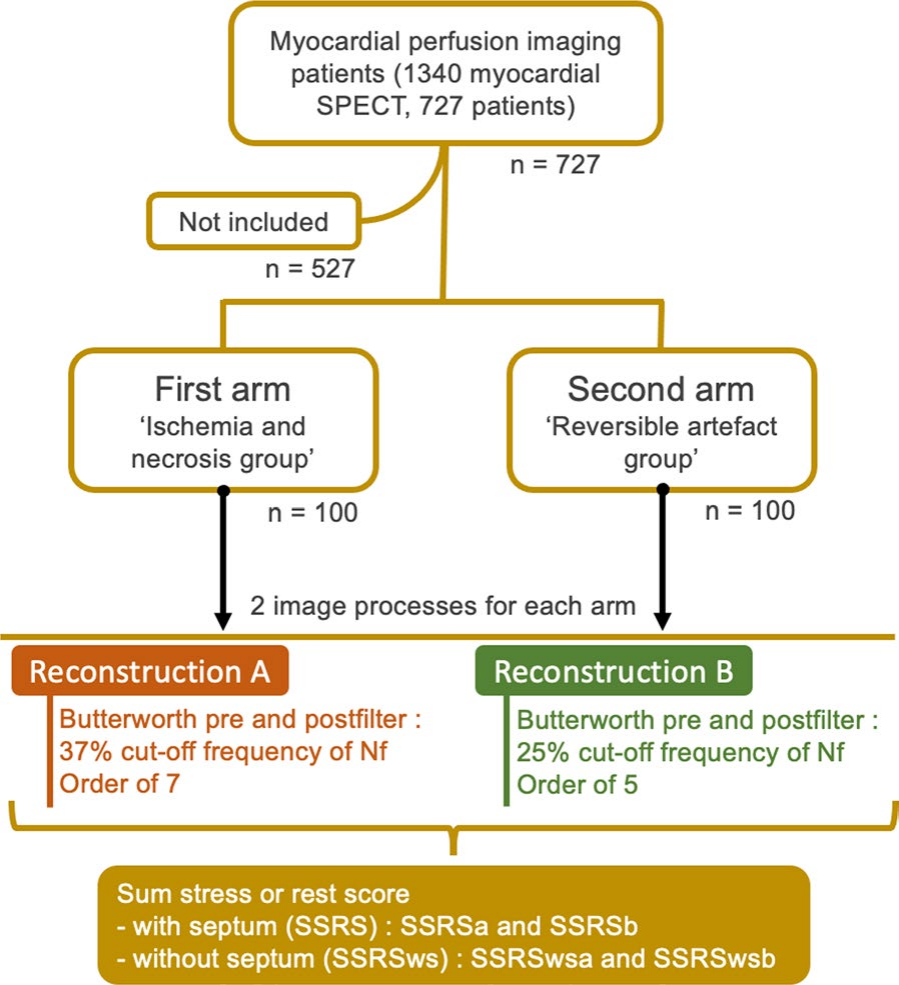
Schematic flow chart of patient’s inclusion, and methods of image processes. SSRS: summed stress or rest score with septum; SSRSws: summed stress or rest score without septum; SPECT: single photon-emission computed tomography

Two arms of 100 patients were prospectively recruited considering stress or rest perfusion SPECT. The schematic flow chart of the inclusion and method is represented in Fig. 1. The recruitment was limited to only one scintigraphy per patient. The first arm named ‘Ischemia and necrosis group’ included all patients with a significant rest or stress perfusion defect (${\text{SSRSws}} \ge 2$), proved to be free from any artefact. All perfusion defects with complete rest reversibility were confirmed by followed coronary angiographic results. All perfusion defects without reversibility were confirmed to be necrotic areas by electrocardiographic, ultrasound computed tomography and cardiologic data. For patients with necrosis diagnosis, rest scintigraphies were selected. For ischemia diagnosis, stress perfusion SPECT were selected. For patients with ischemia associated necrosis, stress perfusion SPECT were selected. For patients with ischemia associated necrosis, coronary angiography was only performed if the ischemia area was over 10% of the myocardial surface. The recruitment for the first arm ended with the analysis of the first 707 patients. The second arm named ‘Reversible artefact group’ included all patients with a heterogeneous stress or rest perfusion SPECT associated to a ${\text{SSRSws}} \ge 2$ proved to be caused by a reversible artefact. This proof was made for the stress SPECT by a strictly normal repeated stress SPECT obtained with repeated acquisition after a delay between 10 and 30 min, and for the rest SPECT with a normal stress SPECT or after repeated acquisition at rest. When artefact occurred for the same patient, for both rest and stress SPECT, the rest scintigraphy was chosen. The recruitment ended with the analysis of 727 patients. Patient with both artefact and ischemia or necrosis diagnosis were not recruited. All non-reversible SPECT artefact were also not selected: all SPECT with perfusion defect caused by a left bundle branch block; all SPECT with body attenuation artefact; all SPECT with parietal digestive activity artefacts considered as non-reversible within the time allowed for the exam.

A first reconstruction process, named reconstruction A, was applied to each acquisition. It corresponds to the default settings of the Discovery NM 530c camera. This reconstruction included a Butterworth prefilter with a cut-off frequency of 0.37*Nf (where Nf stands for Nyquist frequency) and an order of 7, an iterative reconstruction method based on ordered subset expectation maximization (OSEM) with 10 subsets and two iterations, and a Butterworth postfilter with a cut-off frequency of 0.37*Nf and an order of 7. A second reconstruction process, named reconstruction B, was used including a Butterworth prefilter with a cut-off frequency equal to 0.25*Nf and an order equal to 5, an iterative reconstruction method based on OSEM with 10 subsets and two iterations, and a Butterworth postfilter with cut-off frequency equal to 0.25*Nf and an order equal to 5. Four scores were calculated for each included patient: summed stress or rest score with septum (SSRS) and summed stress or rest score without septum (SSRSws) with the reconstruction A (SSRSa and SSRSwsa), and SSRS and SSRSws with the reconstruction B (SSRSb and SSRSwsb). Differences between the two reconstructions were calculated providing two absolute parameters: with septum, $\Delta { } = {\text{SSRSa}} - {\text{SSRSb}}$, and without septum, $\Delta {\text{ws}} = {\text{SSRSwsa}} - {\text{SSRSwsb}}$. Relative differences were normalized on reconstruction A and expressed as: with septum, $\Delta {\text{r}} = \Delta /SSRSa$, and without septum, $\Delta {\text{rws}} = \Delta {\text{ws/}}SSRSwsa$. We used a two-tailed Student t-test to compare the different scores for the two reconstructions. The Bland–Altman plot method was used to compare the two treatments. Statistical significance was defined as $P \le 0.05$.

## Results

Table 1 provides detailed information on patients involved in the study: mean BMI were similar between the two arms while first arm included more men and older patients than the second one. More rest scintigraphies were selected for both arms.

**Table 1:** Characteristics of patients

	First arm	Second arm
n	100	100
Age	66.8 ± 10.8	61.3 ± 11.9
Male	86%	71%
Body mass index	27.4 ± 4.2	28.2 ± 5.3
Rest scintigraphy	68%	70%
Stress scintigraphy	32%	30%

Table 2 shows the quantitative parameters values for the two arms, including SSRSa, SSRSb, SSRSwsa, SSRSwsb, ∆, ∆r, ∆ws, ∆rws, with mean values, standard deviations, and confidence intervals of 95%. Mean SSRS between the two reconstructions varies from 12.8 ± 6.2 to 12.7 ± 6.0 for the first arm (ischemia and necrosis group), and from 6.5 ± 1.9 to 2.0 ± 1.2 for the second arm (reversible artefact group). Mean SSRSws between the two reconstructions varies from 10.7 ± 5.9 to 10.5 ± 5.8 for the first arm, and from 4.5 ± 1.7 to 0.7 ± 0.8 for the second arm. For the first arm, mean SSRS absolute difference between the two reconstructions is evaluated at ∆ = 0.1 ± 1.1 (95% CI − 0.1 to 0.3); and mean SSRS without septal defect absolute difference is ∆ws = 0.2 ± 1.0 (95% CI 0.0–0.4). Both results indicated no-significant differences between the two reconstruction processes for the first arm. Differently, for the second arm, mean SSRS and SSRSws absolute differences between the two reconstruction processes are ∆ = 4.5 ± 1.6 (95% CI 4.2–4.8) and ∆ws = 3.8 ± 1.4 (95% CI 3.6–4.1), respectively. Corresponding relative results are, for the ischemia and necrosis group, ∆r = − 0.5 ± 11.1% (95% CI − 2.7 to 1.7) and ∆rws = 1.3 ± 10.2% (95% CI − 0.7 to 3.3), representing a non-significant difference between the two reconstructions (*P* = 0.54); while, for the reversible artefact group, ∆r = 69.7 ± 16.2% (95% CI 66.6–72.9) and ∆rws = 86.5 ± 14.6% (95% CI 83.6–89.4), representing a significant difference between the two reconstructions (P = 1.7*10^–49^).

**Table 2:** Quantitative parameters result for the two arms

	First arm (n = 100)‘Ischemia and necrosis group’		Second arm (n = 100)‘Reversible artefact group’	
	Mean ± SD	95% CI	Mean ± SD	95% CI
SSRSa	12.8 ± 6.2	[11.5–14.0]	6.5 ± 1.9	[6.1–6.8]
SSRSb	12.7 ± 6.0	[11.5–13.9]	2.0 ± 1.2	[1.7–2.2]
SSRSwsa	10.7 ± 5.9	[9.6–11.9]	4.5 ± 1.7	[4.2–4.8]
SSRSwsb	10.5 ± 5.8	[9.4–11.7]	0.7 ± 0.8	[0.5–0.8]
∆	0.1 ± 1.1	[− 0.1 to 0.3]	4.5 ± 1.6	[4.2–4.8]
∆r (%)	− 0.5 ± 11.1	[− 2.7 to 1.7]	69.7 ± 16.2	[66.6–72.9]
∆ws	0.2 ± 1.0	[0.0–0.4]	3.8 ± 1.4	[3.6–4.1]
∆rws (%)	1.3 ± 10.2	[− 0.7 to 3.3]	86.5 ± 14.6	[83.6–89.4]
SSRSa/SSRSb	*P* = 0.54		*P* = 1.7*10^–49^	
SSRSwsa/SSRSwsb	*P* = 0.05		*P* = 3.3*10^–49^	

*SSRS* summed stress or rest score with septum, *SSRSws* summed stress or rest score without septum, *∆* absolute differences with septum, *∆r* relative differences with septum, *∆ws* absolute differences without septum, *∆rws* relative differences without septum, *P* p-value, *SD* standard deviations, *95% CI* confidence intervals of 95%

**Fig. 2:**
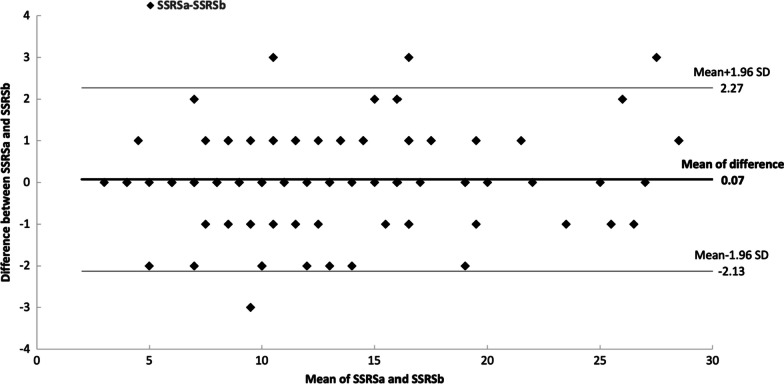
Bland–Altman plot for the first arm summed stress or rest score values with reconstruction **A** and **B**. SSRS: summed stress or rest score with septum; SD: Standard deviation

**Fig. 3:**
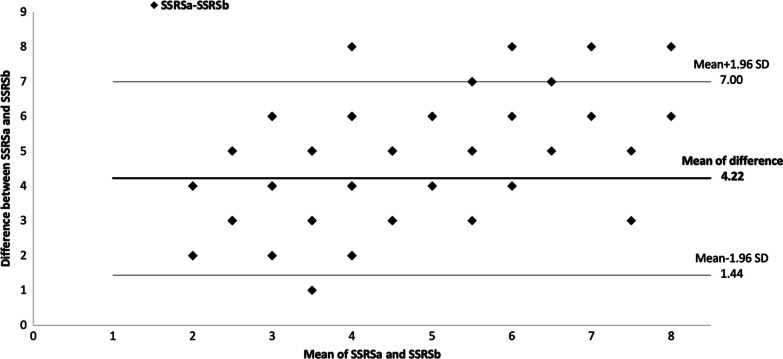
Bland–Altman plot for the second arm summed stress or rest score values with reconstruction **A** and **B**. SSRS: summed stress or rest score with septum; SD: Standard deviation

Bland–Altman plots for the first arm and the second arm are presented in Figs. 2 and 3 respectively. The average difference for the first arm is 0.07. The intervals of agreements comprise 96 out of the 100 patients and range from − 2.13 to 2.27. Because the line of equality is inside the mean difference's confidence interval, the bias is therefore not significant.

The average difference for the second arm is 4.22. The intervals of agreements comprise 95 out of the 100 patients and range from 1.44 to 7.00. Because the line of equality is not inside the mean difference's confidence interval, the bias is therefore significant.

**Fig. 4:**
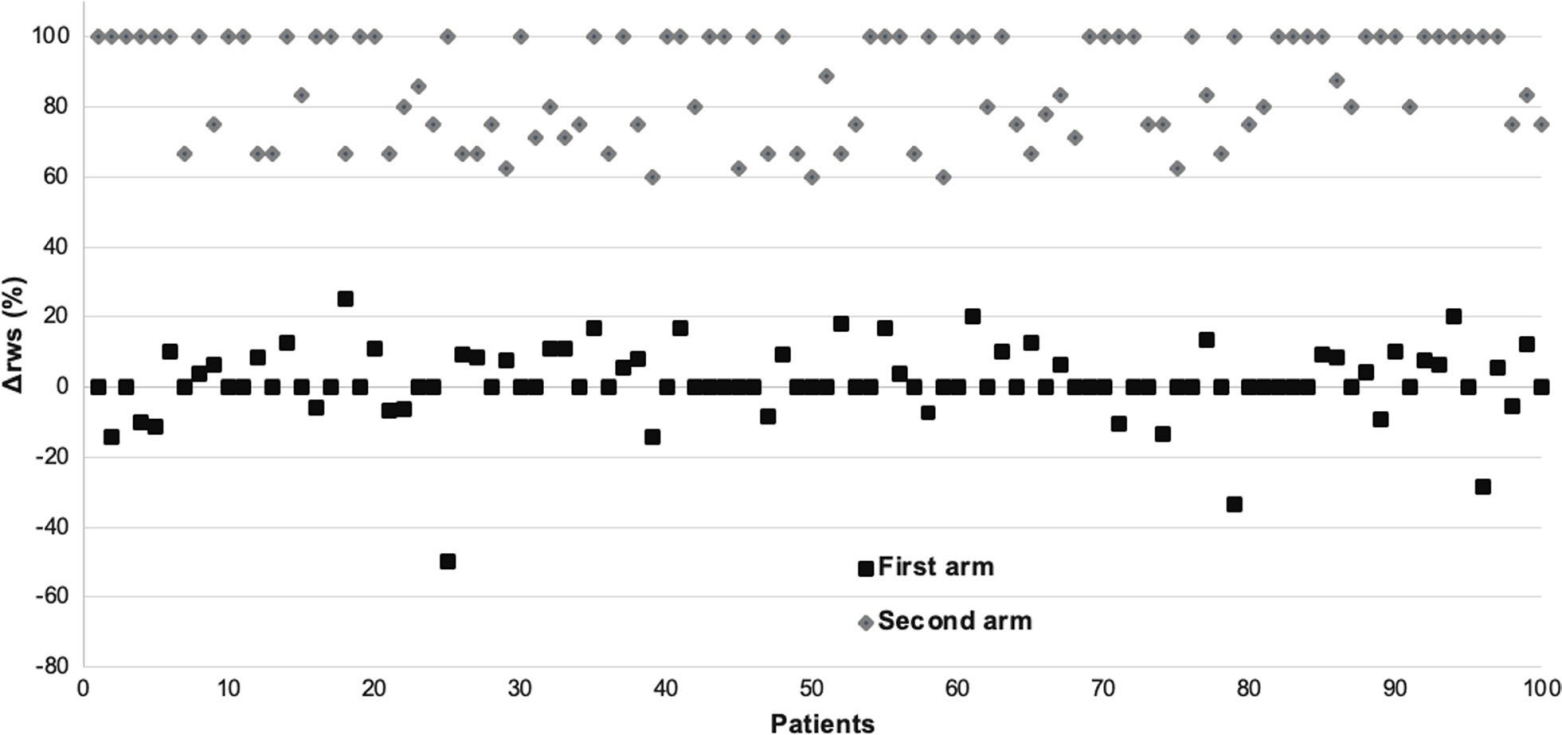
Relative differences in SSRSws between reconstructions **A** and **B** (Δrws) for the two arms. SSRSws: summed stress or rest score without septum; ∆rws: relative differences without septum

The two arms' relative differences in SSRSws between reconstructions A and B (∆rws values) are showed in Fig. 4. We observed two sets of values clearly separated between first arm (ischemia and necrosis group) and second arm (reversible artefact group): in the first arm, ∆rws vary between -50% and 25%; in the second arm ∆rws vary between 60 and 100% without any value lower than 60%.

The first arm was divided into three subgroups discriminating ischemia (n = 17), necrosis (n = 67), and ischemia associated necrosis (n = 16). Table 3 shows the mean quantitative values of the three subgroups. Two-tailed Student t-tests between SSRSa and SSRSb for the subgroups indicate no statistically significant difference for ischemia subgroup, necrosis subgroup, and ischemia associated necrosis subgroup (respectively *P* = 0.79, *P* = 0.67, and *P* = 0.43).

**Table 3:** Quantitative results for the three subgroups of the first arm

	‘Ischemia subgroup’		‘Necrosis subgroup’		‘Ischemia associated necrosis subgroup’	
	Mean ± SD	95% CI	Mean ± SD	95% CI	Mean ± SD	95% CI
Patients (n)	17		67		16	
SSRSa	9.9 ± 6.7	[13.0–6.8]	13.3 ± 6.1	[13.1–13.4]	13.8 ± 5.8	[13.5–14.1]
SSRSb	9.9 ± 6.4	[12.9–7.0]	13.2 ± 6.0	[13.1–13.3]	13.6 ± 5.4	[13.7–14.3]
SSRSwsa	8.1 ± 6.3	[11.0–5.1]	11.1 ± 5.7	[10.9–11.2]	12.1 ± 5.7	[11.8–12.4]
SSRSwsb	8.3 ± 6.3	[11.2–5.4]	10.8 ± 5.6	[10.7–10.9]	11.8 ± 5.5	[11.5–12.0]
∆	− 0.1 ± 0.9	[− 0.5 to 0.4]	0.1 ± 1.2	[0.0–0.1]	0.3 ± 1.2	[0.2–0.3]
∆r (%)	− 1.6 ± 11.9	[− 7.1 to 3.9]	− 0.5 ± 11.5	[− 0.8 to − 0.2]	0.7 ± 8.5	[0.3–1.2]
∆ws	− 0.2 ± 1.0	[− 0.7 to 0.2]	0.3 ± 0.9	[0.2–0.3]	0.3 ± 1.2	[0.3–0.4]
∆rws (%)	− 3.6 ± 15.9	[− 10.9 to 3.7]	2.1 ± 8.4	[1.9–2.3]	3.2 ± 8.8	[2.8–3.7]
SSRSa/SSRSb	*P* = 0.79		*P* = 0.67		*P* = 0.43	

*SSRS* summed stress or rest score with septum, *SSRSws* summed stress or rest score without septum, *∆* absolute differences with septum, *∆r* relative differences with septum, *∆ws* absolute differences without septum, ∆rws relative differences without septum, *P* p-value, SD standard deviations, *95% CI* confidence intervals of 95%

For 83 patients, ∆ are equal to − 1, 0 or 1, meaning the second reconstruction does not change significantly the SSRS. For 10 patients, ∆ are negative, equal to − 2 or -3, meaning a higher SSRS in these cases and no sensibility loss. For 7 patients, ∆ are equal to 2 or 3, with 5 patients with necrosis and 2 patients with ischemia associated necrosis. For these patients, the relative difference is 7% < ∆r < 25%.

The distribution of the remaining SSRSws after reconstruction B for the second arm is showed in Table 4a. We found that 85% of patients present a non-significant SSRSwsb inferior or equal to 1. SSRSwsb remaining value is 3 for 2 patients, with a relative difference Δrws of 63%, not considered as normal.

**Table 4:** (a) Distribution of the remaining SSRSws after reconstruction B for the second arm; (b) MPI’s characteristics of the patients with a remaining SSRSws equals to 2

a				
Second arm’s SSRSwsb	0	1	2	3
Patients (n = 100) %	50%	35%	13%	2%

b					
Patient (n = 13)	SPECT	SSRSws	Value (%)	Value (%)	Territory
1	Rest	2	65	67	None
2	Rest	2	66	69	None
3	Rest	2	68	69	None
4	Rest	2	61	69	Inferior
5	Stress	2	60	66	Inferior
6	Stress	2	65	65	Inferior
7	Rest	2	64	69	None
8	Rest	2	62	66	Inferior
9	Rest	2	68	69	Inferior
10	Stress	2	69	69	None
11	Rest	2	65	69	None
12	Rest	2	57	68	Inferior
13	Rest	2	68	68	Inferior

*SSRSws* summed stress or rest score without septum, *SPECT* single photon-emission computed tomography

In Table 4b are presented MPI’s characteristics of the 13 patients with a remaining SSRSws equals to 2, including stress or rest acquisition, with a relative difference Δrws after the second reconstruction between 60 and 75%. The segmental perfusion defects' values are shown, as well as the myocardial territory when the defects are close to segments. A supplemental study of the perfusion defects indicates 26 abnormal segments, with a percentage value superior or equal to 65% for 21 segments, and a mean value of 66.2%. For 7 out of the 13 MPI images with a remaining SSRSws equals to 2, the only territory concerned is the inferior: 2 of them have defect values of 68% and 69%, considered as normal; and 5 of them (2 stress and 3 rest SPECT) not considered as normal. For the 6 others, MPI defects concern other territories, considered as normal.

In total, 7 out of 100 patients had images not considered as normal after reconstruction B.

**Fig. 5:**
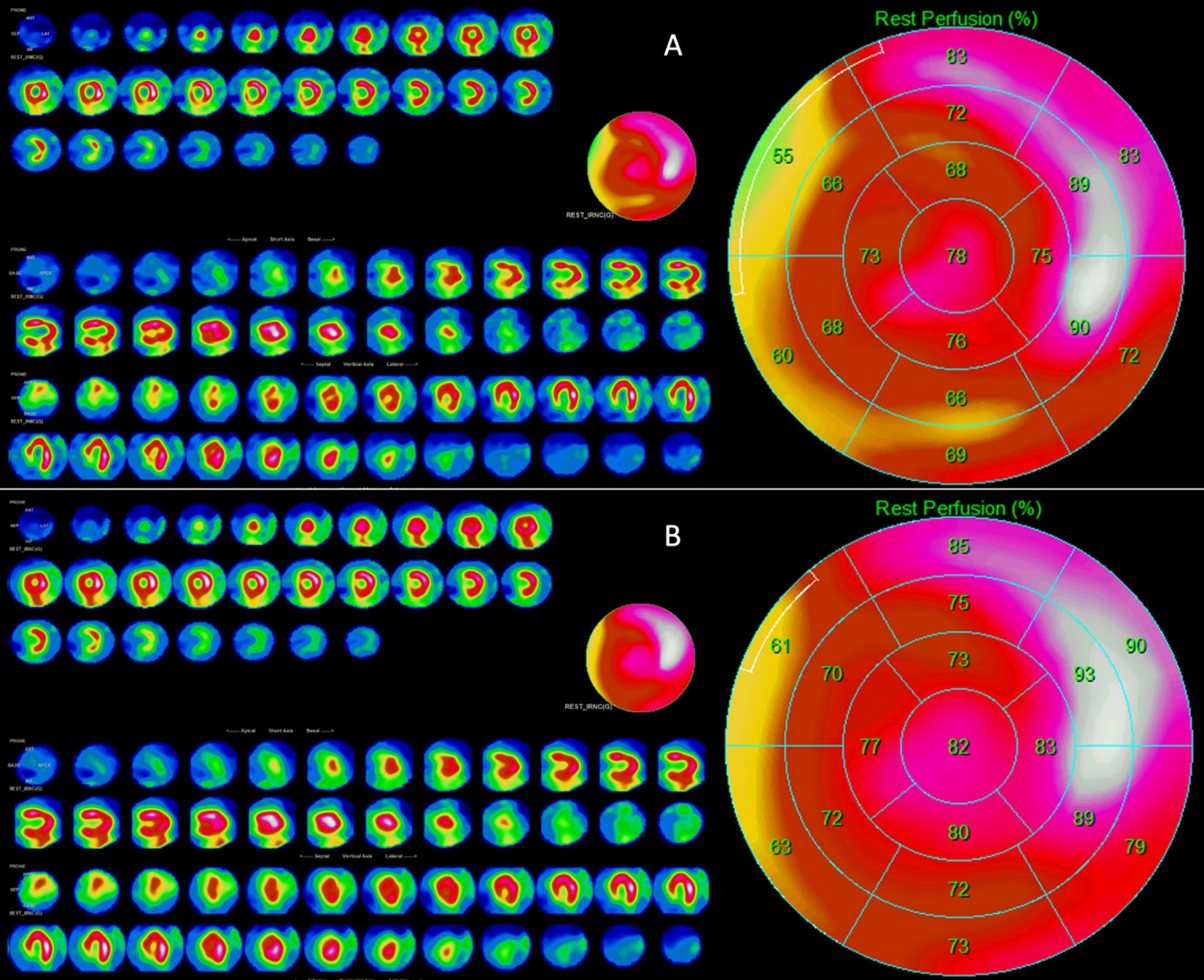
Representative images of reconstructions **A** and **B** on the same scintigraphy acquisition. Summed stress or rest score without septum after reconstruction **A** (SSRSwsa) = 5; Summed stress or rest score without septum after reconstruction **B** (SSRSwsb) = 0; ∆ws = 5; ∆rws = 100%

Figure 5 shows representative images demonstrating the impact of the new filtering approach on image quality. After applying reconstruction process B, the SSRSws score decreased from 5 to 0, reflecting an artefact generated by reconstruction process A.

## Discussion

During the study, 727 patients were explored, 1340 myocardial SPECT were performed, and 100 reversible SPECT artefacts were selected. The latter represent 13.7% of the 727 patients, considering that only one scintigraphy was analysed for a single patient.

Regarding the first arm, two-tailed Student t-test showed no significant statistical difference between reconstruction A and B. All the parameters investigated, relative or absolute, ∆, ∆r, ∆ws and ∆rws show no-significant differences between reconstruction A and B, indicating that the evaluation of ischemia or necrosis is strictly identical with the two reconstructions. For all the patients with necrosis, diagnosis is unchanged. Both the diagnosis and the ischemia’s extent are identical with the two reconstructions for the two patients who have ischemia-associated necrosis. Subgroups analysis indicates no significant statistical difference between the three subgroups using SSRSa and SSRSb. These results confirm that the evaluation of the SSRS is strictly identical for ischemia and necrosis with reconstruction A or reconstruction B.

For the second arm, the SSRS obtained with the two reconstructions were significantly different with a ∆r relative difference of 69.7 ± 16.2%. A score of SSRSwsb that is not different from 0 indicates that reconstruction B generates no MPI defect for patient scintigraphy initially concerned by significant artefact with reconstruction A. For the 70 rest SPECT with artefacts of the second arm, 21 were identified with strictly normal repeated acquisition at rest after delay and 49 were identified with strictly normal acquisition after stress. Remaining values of SSRSwsb indicates an average of 0.7 ± 0.8 (95% CI 0.5–0.8) meaning that without non-specific septal heterogeneity, SSRS mean value can be considered of 0 or 1, i.e., non-significant. Finally, 93 of the 100 scintigraphies can be considered normal after the second reconstruction, meaning that reconstruction B generates normal SPECT without artefact for 93% of the recruited patients. Reconstruction B could enhance first rest or stress SPECT specificity by 12.8% at the most, according to the 93/100 reversible artefacts found on out 727 patients.

Two-tailed Student t-test showed no significant statistical difference between the two arms BMI (*P* = 0.26). The first arm included older patients with a bigger number of men. These results can be explained by the increase of coronary pathology risk with age and the masculine gender, which also has a higher BMI in general. The differences between our two arms represent no significant bias because they’re not directly compared.

The filters modifications from reconstruction process A (0.37*Nf) to reconstruction process B (0.25*Nf) are not a simple Gaussian filter applied to final results, because they occurred before iterative reconstruction. In other words, the reconstruction process B does not correct reversible artefacts but does not generate these artefacts through OSEM iterative reconstruction. Artefacts that are not generated do not require to be corrected. In fact, the optimized filters strategy may prevent the iterative reconstruction to generate heterogeneous solutions with false hypoperfusions as confirmed by normalisation after repeated acquisitions. These artefacts reduce the scintigraphy specificity that in the worst cases can lead to inappropriate coronary angiography and extend in all cases the medical care duration with repeated acquisitions. In addition, reconstruction B could be a valuable strategy to optimize all patients’ medical care. The reconstruction process B with adjusted Butterworth prefilter and post-filter parameters allows to improve the specificity and to preserve the sensibility compared to the reconstruction process A. Also, a relative difference Δrws superior to 60% with a remaining SSRS after reconstruction B inferior or equal to 2 indicates a reversible artefact in case of heterogeneous acquisition. In cases of ischemia and necrosis, hypoperfusion remains after applying the reconstruction B with relative difference Δrws inferior to 20%. Intermediate relative difference with Δrws between 20 and 60% can occur but in cases of left bundle branch block and body attenuation artefacts. These cases were not recruited in our study because they generate non-reversible artefacts.

## Conclusion

This study proposes a simple method to identify reversible artefacts in MPI SPECT without repeated acquisitions. These results support the theory that reconstruction B, in comparison to conventional reconstruction A, generates fewer artefact defects and does not alter SSRS for authentic defects. Enhanced filters strategy of reconstruction B allows to increase MPI specificity by 12.8% without changing the sensibility. This reconstruction may be used as an additional method that allows to discriminate reversible artefact from ischemia and necrosis. This strategy also optimizes coronary angiography explorations and patient medical care, reducing number of false positive results and number of repeated SPECT in cases of heterogeneous scintigraphy. A complementary strategy may propose the exploration of the myocardial acquisition with the two filters (reconstruction A and B) to directly discriminate ischemia or necrosis from reversible artefacts using Δrws parameter.

## Data Availability

The datasets generated during and/or analysed during the current study are available from the corresponding author on reasonable request.

## References

[1] Imbert L, Poussier S, Franken PR, Songy B, Verger A, Morel O, Wolf D, Noel A, Karcher G, Marie PY (2012). Compared performance of high-sensitivity cameras dedicated to myocardial perfusion SPECT: a comprehensive analysis of phantom and human images. J Nucl Med.

[2] Imbert L, Marie PY (2016). CZT cameras: a technological jump for myocardial perfusion SPECT. J Nucl Cardiol.

[3] Zoccarato O, Lizio D, Savi A, Indovina L, Scabbio C, Leva L, Del Sole A, Marcassa C, Matheoud R, Lecchi M, Brambilla M (2016). Comparative analysis of cadmium-zincum-telluride cameras dedicated to myocardial perfusion SPECT: a phantom study. J Nucl Cardiol.

[4] Erlandsson K, Kacperski K, van Gramberg D, Hutton BF (2009). Performance evaluation of D-SPECT: a novel SPECT system for nuclear cardiology. Phys Med Biol.

[5] Agostini D, Marie PY, Ben-Haim S, Rouzet F, Songy B, Giordano A, Gimelli A, Hyafil F, Sciagrà R, Bucerius J, Verberne HJ, Slart RH, Lindner O, Übleis C, Hacker M, Cardiovascular Committee of the European Association of Nuclear Medicine (EANM) (2016). Performance of cardiac cadmium-zinc-telluride gamma camera imaging in coronary artery disease: a review from the cardiovascular committee of the European Association of Nuclear Medicine (EANM). Eur J Nucl Med Mol Imaging.

[6] Nudi F, Iskandrian AE, Schillaci O, Peruzzi M, Frati G, Biondi-Zoccai G (2017). Diagnostic accuracy of myocardial perfusion imaging with CZT technology: systemic review and meta-analysis of comparison with invasive coronary angiography. JACC Cardiovasc Imaging.

[7] Cantoni V, Green R, Acampa W, Zampella E, Assante R, Nappi C, Gaudieri V, Mannarino T, Cuocolo R, Di Vaia E, Petretta M, Cuocolo A (2019). Diagnostic performance of myocardial perfusion imaging with conventional and CZT single-photon emission computed tomography in detecting coronary artery disease: a meta-analysis. J Nucl Cardiol.

[8] Lima R, Peclat T, Soares T, Ferreira C, Souza AC, Camargo G (2017). Comparison of the prognostic value of myocardial perfusion imaging using a CZT-SPECT camera with a conventional anger camera. J Nucl Cardiol.

[9] Gimelli A, Bottai M, Giorgetti A, Genovesi D, Filidei E, Marzullo P (2012). Evaluation of ischaemia in obese patients: feasibility and accuracy of a low-dose protocol with a cadmium-zinc telluride camera. Eur J Nucl Med Mol Imaging.

[10] Oddstig J, Martinsson E, Jögi J, Engblom H, Hindorf C (2018). Differences in attenuation pattern in myocardial SPECT between CZT and conventional gamma cameras. J Nucl Cardiol.

[11] Kennedy JA, Brodov Y, Weinstein AL, Israel O, Frenkel A (2019). The effect of CT-based attenuation correction on the automatic perfusion score of myocardial perfusion imaging using a dedicated cardiac solid-state CZT SPECT/CT. J Nucl Cardiol.

[12] Moncayo VM, Galt J. Attenuation correction in multipinhole-CZT gamma camera: Differences in attenuation pattern in myocardial SPECT between CZT and conventional gamma cameras. Oddstig J, Martinsson E, Jogi J, Engblom H, Hindorf C. J Nucl Cardiol. 2018. J Nucl Cardiol. 2019; 10.1007/s12350-018-01498-7 10.1007/s12350-018-01498-7 30465233

[13] van Dijk JD, Mouden M, Ottervanger JP, van Dalen JA, Knollema S, Slump CH, Jager PL (2017). Value of attenuation correction in stress-only myocardial perfusion imaging using CZT-SPECT. J Nucl Cardiol.

[14] Daou D, Sabbah R, Coaguila C, Boulahdour H (2018). Impact of data-driven cardiac respiratory motion correction on the extent and severity of myocardial perfusion defects with free-breathing CZT SPECT. J Nucl Cardiol.

[15] Redgate S, Barber DC, Fenner JW, Al-Mohammad A, Taylor JC, Hanney MB, Tindale WB (2016). A study to quantify the effect of patient motion and develop methods to detect and correct for motion during myocardial perfusion imaging on a CZT solid-state dedicated cardiac camera. J Nucl Cardiol.

[16] Van Dijk JD, van Dalen JA, Mouden M, Ottervanger JP, Knollema S, Slump CH, Jager PL (2018). Value of automatic patient motion detection and correction in myocardial perfusion imaging using a CZT-based SPECT camera. J Nucl Cardiol.

[17] Clerc OF, Fuchs TA, Possner M, Vontobel J, Mikulicic F, Stehli J, Liga R, Benz DC, Gräni C, Pazhenkottil AP, Gaemperli O, Buechel RR, Kaufmann PA (2017). Real-time respiratory triggered SPECT myocardial perfusion imaging using CZT technology: impact of respiratory phase matching between SPECT and low-dose CT for attenuation correction. Eur Heart J Cardiovasc Imaging.

[18] Hindorf C, Oddstig J, Hedeer F, Hansson MJ, Jögi J, Engblom H (2014). Importance of correct patient positioning in myocardial perfusion SPECT when using a CZT camera. J Nucl Cardiol.

